# Expression of p15^INK4b^ and p57^KIP2^ and Relationship with Clinicopathological Features and Prognosis in Patients with Vulvar Squamous Cell Carcinoma

**DOI:** 10.1371/journal.pone.0061273

**Published:** 2013-04-08

**Authors:** Ruth Holm, Mette Førsund, Mai T. Nguyen, Jahn M. Nesland, Claes G. Trope

**Affiliations:** 1 Department of Pathology, The Norwegian Radium Hospital, Oslo University Hospital, Oslo, Norway; 2 Department of Pathology, The Norwegian Radium Hospital, Oslo University Hospital and University of Oslo, Oslo, Norway; 3 Department of Obstetrics and Gynecology, The Norwegian Radium Hospital, Oslo University Hospital and University of Oslo, Oslo, Norway; Moffitt Cancer Center, United States of America

## Abstract

**Background:**

The cyclin-dependent kinase inhibitors p15^INK4b^ and p57^KIP2^ are important regulators of the cell cycle, and their abnormal expression has been detected in various tumors. However, little is known about the role of p15^INK4b^ and p57^KIP2^ in the pathogenesis of vulvar carcinoma, and the prognostic impact is still unknown. In our current study, we examined the expression of p15^INK4b^ and p57^KIP2^ in a large series of vulvar squamous cell carcinomas to elucidate the prognostic impact.

**Methods:**

Expression of p15^INK4b^ and p57^KIP2^ were examined in 297 vulvar squamous cell carcinomas using immunohistochemistry. Both uni- and multivariate analysis of prognostic factors were performed, and correlations with clinicopathologic parameters were examined.

**Results:**

Compared to the high levels of p15^INK4b^ and p57^KIP2^ in normal vulvar squamous epithelium, low levels of p15^INK4b^ and p57^KIP2^ were found in 82% and 44% of vulvar carcinomas, respectively. Low levels of p15^INK4b^ and p57^KIP2^ correlated significantly with malignant features, including large tumor diameter (*p* = 0.03 and *p* = 0.001, respectively) and increased invasiveness (*p* = 0.003 and *p* = 0.04, respectively). Although p15^INK4b^ and p57^KIP2^ levels could not be identified as prognostic markers, combined analysis of p14^ARF^/p15^INK4b^/p16^INK4a^ showed that patients whose tumors expressed low levels of two or three of these INK4 proteins had a worse prognosis than those with only low levels of one or no protein (univariate analysis *p* = 0.02). The independent prognostic significance of these INK4 proteins was confirmed by multivariate analysis (*p* = 0.008).

**Conclusions:**

We show for the first time that p15^INK4b^ and p57^KIP2^ may be involved in the progression of vulvar carcinomas and the combined p14^ARF^/p15^INK4b^/p16^INK4a^ status was a statistically independent prognostic factor.

## Introduction

Vulvar carcinoma is a rare female genital malignancy with an incidence ranging from 1 to 2 per 100 000 person-years worldwide [1], [2]. Vulvar cancer has mainly been linked to elderly women but an increasing incidence among younger women has been reported recently [3], [4]. The standard treatment with radical surgery is associated with a considerable morbidity [1]. Thus, it is important to make individualized treatment procedures in order to reduce negative effects for patients with a good prognosis. The identification of new biomarkers could possibly improve the prediction of clinical outcome and may also be important for development of better treatment strategies.

Cyclin-dependent kinase inhibitors (CDKI), the major inhibitors of the cell cycle, are divided into the INK4 and CIP/KIP family. The INK4 members includes p15^INK4b^, p16^INK4a^, p18^INK4c^ and p19^INK4d/ARF^ (p14^ARF^ in humans), whereas the CIP/KIP members includes p21^CIP1^, p27^KIP2^ and p57^KIP2^ [5], [6]. INK4 protein binds specifically to the CDK4 and CDK6 complexes, causing G1 arrest. The CIP/KIP family has a broader specificity for CDKs also inhibiting the other cyclin-CDK complexes at a later stage of the cell cycle [5], [6]. Previously p14^ARF^, p15^INK4b^, p16^INK4a^, p21^CIP1^ and p27^KIP2^ have been found to be involved in the neoplastic process of vulvar carcinomas [7]–[9], however only a limited number of cases has been investigated for p15^INK4b^ [9]. Furthermore, to our knowledge, there has been no study of p57^KIP2^ in vulvar carcinomas. The loss of p15^INK4b^ and p57^KIP2^ function occurs frequently in a variety of human cancers suggesting that its down-regulation may be important in neoplastic transformation [10]–[18]. Furthermore, an association has been found between abnormality of p15^INK4b^ and p57^KIP2^ and unfavorable outcome [14], [17], [19]–[23]. The aim of our study was to investigate the expression of p15^INK4b^ and p57^KIP2^ in a large series of vulvar squamous cell carcinomas to clarify their potential prognostic values.

## Methods

### Patient materials

Between 1977 and 2006, 297 patients had been diagnosed with vulvar squamous cell carcinoma at The Norwegian Radium Hospital. The median age at diagnosis was 74 years (range 35–96 years). Surgical radicality in the vulvar specimen (no rest tumor) was obtained in 198 (67%) of the patients and the remaining 99 (33%) patients did not obtain surgical radicality. Prior to surgery, radiotherapy was given to six patients and three cases received radiotherapy/chemotherapy. Postoperative irradiation was administered to 63, chemotherapy to three and irradiation/chemotherapy to four of the patients. After treatment, the patients have been followed at The Norwegian Radium Hospital or at a local hospital. Follow-up information is available for all patients until 1. September, 2009. During follow-up, 122 (40%) patients died of vulvar cancer. The median follow-up time for patients still alive was 151 months (range, 43 to 378 months). The tumors were all staged based on the International Federation of Gynecology and the Obstetrics (FIGO) classification from 2009 [24]. The Regional Committee for Medical Research Ethics South of Norway (S-06012), The Social and Health Directorate (04/2639 and 06/1478) and The Data Inspectorate (04/01043) approved the current study protocol. In this study we have used paraffin embedded tumor tissue from vulvar cancer patients diagnosed between 1977 and 2006. Many of these patients are either dead or very old. Therefore, we have not been able to obtain patient consent. Permission has been obtained from The Social and Health Directorate (04/2639) to perform this study without patient consent.

The histological specimens were reevaluated by an experienced pathologist (J.M.N) according to World Health Organization recommendations [25]. Two hundred and eighty (94%) tumors were keratinizing/nonkeratinizing, 13 (5%) were basaloid and 4 (1%) were veruccoid. Thirty-six samples of normal vulva form patients, undergoing surgery for benign gynecological diseases, were included as controls. Results obtained from our previous studies on cell cycle proteins in this same cohort of vulvar carcinomas [7], [8], [26] were co-analyzed with those of the current study.

### Immunohistochemistry

Sections from formalin-fixed, paraffin-embedded tissues were immunostained using the Dako EnVision™ Flex+ System (K8012; Dako, Glostrup, Denmark) and the Dako Autostainer. Deparaffinization and the unmasking of epitopes were performed using PT-Link (Dako) and EnVision™ Flex target retrieval solution at a high pH for p15^INK4b^ and low pH for p57^KIP2^. To block endogeneous peroxidase the sections were treated with 0.3% hydrogen peroxide (H_2_O_2_) for 5 min. Sections were incubated overnight at 4°C with monoclonal antibody p15^INK4b^ (clone 15P06, 1∶500, 0.4 µg IgG_1_/ml, Thermo Fischer Scientific, Fremont, CA, USA) and polyclonal rabbit antibody p57^KIP2^ (1∶1000, 0.14 µg IgG/ml, Sigma, St. Lous, MO, USA). The specimens were subsequently treated with goat anti-mouse IgG or goat anti-rabbit IgG for 30 min, EnVision™ Flex/HRP enzyme for 30 min, 3′3-diaminobenzidine tetrahydrochloride (DAB) for 10 min, counterstained with hematoxylin, dehydrated and mounted in Richard-Allan Scientific Cyto seal XYL (Thermo Scientific, Waltham, MA, USA). All of the sample series included appropriate positive controls, which included normal vulva (p15^INK4b^) and ovarian serous cystadenoma (p57^KIP2^). Negative controls included substitution of the monoclonal antibody with mouse myceloma protein of the same subclass and concentration as the monoclonal antibody, or normal rabbit IgG of the same concentration as the polyclonal antibody.

Semiquantitative classes were used to describe the extent of staining (percent of positive tumor cells: absent, 0; <10%, 1; 10–50%, 2; >50%, 3) and intensity (absent, 0; weak, 1; moderate, 2; strong, 3). By multiplying the extent and the intensity of the signal, product scores for nuclear staining were produced which ranging from 0 to 9. Protein levels for p15^INK4b^ and p57^KIP2^ were classified as high when a score of 9 and low when a score of <9. The cutoff value for the immunoreactivity was based on staining pattern observed in normal vulvar epithelium. Examination of immunostaining was performed in a blinded fashion by two observers (R.H. and J.M.N) with no knowledge of the clinicopathological variables, patient outcomes and cell cycle protein results from our previous studies [7], [8], [26]. All discordant scores were reviewed until a final agreement was obtained.

### Statistical analyses

The Pearson's chi-square (χ^2^) and Linear-by-linear association were performed to fine associations between protein expression and clinicopathologic variables. Survival analysis was processed using the Kaplan and Meier estimation and log-rank test. Disease-specific survival was calculated from the date of diagnosis to vulvar cancer related death. A Cox proportional hazards regression model was used for both univariate and multivariate evaluation of survival rates. In the multivariate analysis, a backward stepwise regression was performed with a *p*≤0.05 as the inclusion criterion for variables in the univariate analysis. All calculations were processed using SPSS 18.0 statistical software package (SPSS, Chicago, IL, USA) and statistical significance was considered as *p*≤0.05.

The vulvar carcinoma tissues in our cohort have been collected over an extensive period from 1977–2006. Due to the large variation in storage time and given that the fixation protocol for these tissues up to 1987 was acid formalin, whereas from 1987–2006 was buffered formalin, Mann-Whitney U test was used to evaluate whether this has any influence on the p15^INK4b^ and p57^KIP2^ immunostaining. The Mann-Whitney U test showed that the distribution of p15^INK4b^ and p57^KIP2^ expression was the same between samples processed before and after 1987.

## Results

**Figure 1:**
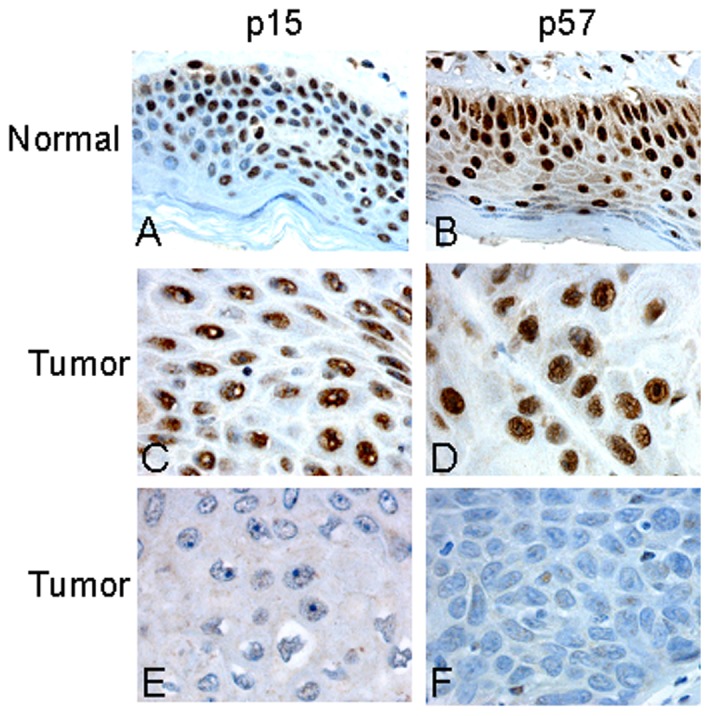
Immunohistochemical staining of p15^INK4b^ and p57^KIP2^ protein in vulvar squamous epithelium. High nuclear expression of p15^INK4b^ (A) and p57^KIP2^ (B) in normal vulvar epithelium. High nuclear expression of p15^INK4b^ (C) and p57^KIP2^ (D) and low nuclear expression of p15^INK4b^ (E) and p57^KIP2^ (F) in vulvar carcinomas.

In 36 cases of normal vulvar squamous epithelium, nuclear staining for p15^INK4b^ and p57^KIP2^ was identified in basal, parabasal, middle and top layers with a score of 9 (Figure 1A and B). The immunostaining results in vulvar carcinomas are summarized in Table 1. High p15^INK4b^ and p57^KIP2^ immunostaining (score 9) in the nucleus was observed in 53/297 (18%) and 165/297 (56%) of the cases, respectively (Figure 1C–D), whereas low expression (score <9) was identified in 244/297 (82%) and 132/297 (44%), respectively (Figure 1E–F).

**Table 1:** Immunostaining results for p15^INK4b^ and p57^KIP2^.

Score	p15^INK4b^		p57^KIP2^	
	N	(%)	N	(%)
0	28	(9.4)	1	(0.3)
1	6	(2.0)	1	(0.3)
2	38	(12.8)	1	(0.3)
3	23	(7.7)	7	(2.4)
4	61	(20.5)	20	(6.7)
6	88	(29.6)	102	(34.3)
9	53	(17.8)	165	(55.6)
Total	297	(100.0)	297	(100.0)

The levels of p15^INK4b^ and p57^KIP2^ in relation to clinicopathological parameters are shown in Table 2. Low expression of p15^INK4b^ and p57^KIP2^ were significantly correlated with large tumor diameter (*p* = 0.03 and *p* = 0.001, respectively) and deep invasion (*p* = 0.003 and *p* = 0.04, respectively). In addition, low expression of p57^KIP2^ significantly correlated with younger age (*p* = 0.03) and a low level of cyclin D3 (*p* = 0.05).

**Table 2:** p15^INK4b^ and p57^KIP2^ expression in relation to clinicopathological variables.

Variables	Total	p15^INK4b^				p57^KIP2^			
	N	High	Low (%)		*p*	High	Low (%)		*p*
Age					0.15^1^				**0.03** 1
25–69	117	26	91	(78)		57	60	(51)	
70–84	146	22	124	(85)		85	61	(42)	
85+	34	5	29	(85)		23	11	(32)	
FIGO					0.30^2^				0.11^2^
Ia	10	1	9	(90)		7	3	(30)	
Ib	137	32	105	(77)		82	55	(40)	
II	13	1	12	(92)		3	10	(77)	
IIIa	64	11	53	(83)		31	33	(52)	
IIIb	38	4	34	(89)		24	14	(37)	
IIIc	12	1	11	(92)		8	4	(33)	
IVa	5	0	5	(100)		3	2	(40)	
IVb	13	3	10	(77)		5	8	(62)	
Not available	5								
Lymph node metastasis					0.18^2^				0.73^2^
None	164	35	129	(79)		95	69	(42)	
Unilateral	89	13	76	(85)		47	42	(47)	
Bilateral	38	4	34	(89)		21	45	(45)	
Not available	6								
Tumor diameter (cm)					**0.03** 1				**0.001** 1
0.3–2.5	88	22	66	(75)		61	27	(31)	
2.6–4.0	93	15	78	(84)		50	43	(46)	
4.1–20.0	100	13	87	(87)		44	56	(56)	
Not available	16								
Tumor differentiation					0.30^2^				0.06^2^
Well	73	12	61	(84)		48	25	(34)	
Moderate	153	32	121	(79)		84	69	(45)	
Poor	71	9	62	(87)		33	38	(53)	
Depth of invasion (mm)					**0.003** 1				**0.04** 1
0.0–4.0	76	23	53	(70)		49	27	(36)	
4.1–8.0	98	14	84	(86)		55	43	(44)	
8.1–40.0	112	14	98	(88)		55	57	(51)	
Not available	11								
Infiltration of vessel					0.86^2^				0.61^2^
No	229	41	188	(82)		128	101	(44)	
Yes	65	11	54	(83)		34	31	(48)	
Not available	3								

1 Linear-by-linear association.

2 Pearson chi-square.

High: Expression in nucleus = 9.

Low: Expression in nucleus <9.

**Figure 2:**
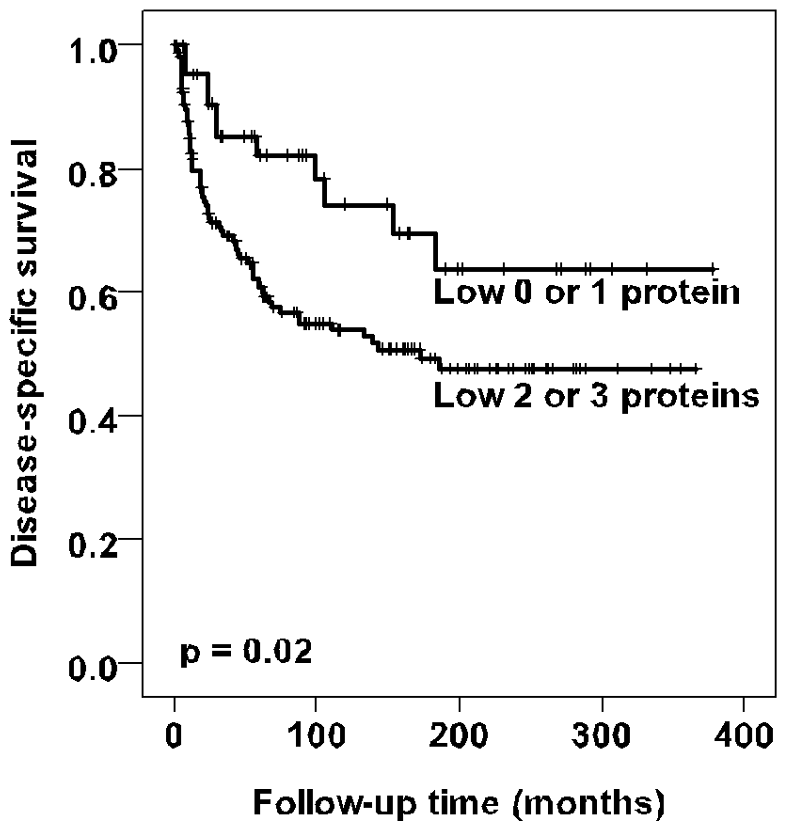
Survival curves using the Kaplan-Meier method. The Kaplan-Meier curve of disease-specific survival in relation to the combined analysis of p14^ARF^/p15^INK4b^/p16^INK4a^ showed that patients whose tumors expressed low levels of two or three of these INK4 proteins had a worse prognosis than those with only low levels of one or no protein (*p* = 0.02).

By univariate analysis neither p15^INK4b^ nor p57^KIP2^ were associated with disease-specific survival (*p* = 0.29 and *p* = 0.94). Because the prognosis of patients most likely do not dependent on the expression of one single member of the INK4 and CIP/KIP families but on an interplay between the different proteins, the previously determined p16^INK4a^ and p14^ARF^ status [7], [8] were combined with the expression of p15^INK4b^ and the previously identified p27^KIP1^ and p21^CIP1^ data [7] were combined with the expression of p57^KIP2^ for each patient. Patients whose tumors expressed low levels of two or three of these INK4 proteins have a worse prognosis than those with only low levels of one or no protein (*p* = 0.02) (Figure 2). Hovewer, the combination of the CIP/KIP members were not significantly correlated to clinical outcome (*p* = 0.78). By multivariat analysis, lymph node metastasis, vessel infiltration, tumor diameter and the combined p14^ARF^/p15^INK4b^/p16^INK4a^ status were of independent prognostic significance (Table 3).

**Table 3:** Relative risk (RR) of dying from vulvar cancer.

Variables	Univariate analysis			Multivariate analysis		
	RR	95% CI^a^	*p*	RR	95% CI^a^	*p*
Lymph node metastasis	2.33	1.85–2.94	<0.001	1.74	1.28–2.36	<0.001
Infiltration of vessel	2.46	1.68–3.60	<0.001	2.78	1.71–4.52	<0.001
Tumor diameter	1.74	1.38–2.20	<0.001	1.59	1.18–2.15	0.003
p14^ARF^/p15^INK4b^/p16^INK4a^ combined (0+1 *vs* 2+3)^b^	2.12	1.12–4.01	0.02	2.50	1.27–4.90	0.008
p14^ARF^/p15^INK4b^/p16^INK4a^ combined (0+1 *vs* 2+3)^b^^,^ c	2.22	0.95–5.18	0.06	2.67	1.04–6.87	0.04

a 95% confidence interval.

b 0+1: 0 or 1 protein with low expression; 2+3: 2 or 3 proteins with low expression.

c Subgroup of patients obtained surgical radicality (no rest tumor).

**Figure 3:**
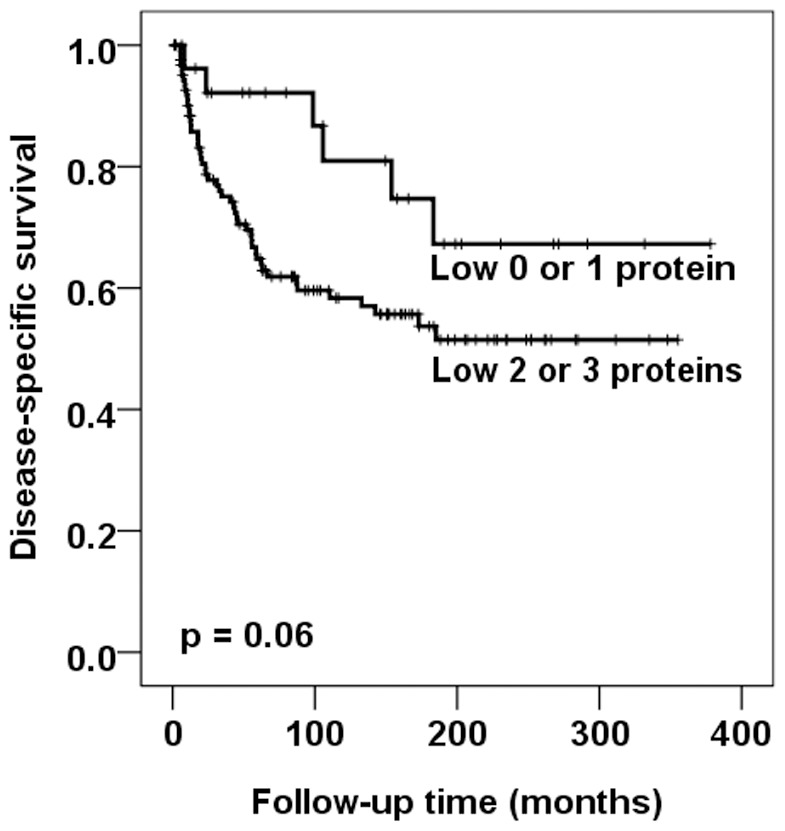
Survival curves using the Kaplan-Meier method for a subgroup of patients obtained surgical radicality (no rest tumor). The Kaplan-Meier curve of disease-specific survival in relation to the combined analysis of p14^ARF^/p15^INK4b^/p16^INK4a^ showed that patients whose tumors expressed low levels of two or three of these INK4 proteins had a worse prognosis than those with only low levels of one or no protein (*p* = 0.06).

The results from the univariate analysis combining p14^ARF^/p15^INK4b^/p16^INK4a^ status in a subgroup of patients who obtained surgical radicality (no rest tumor) is showed in Figure 3. In this subgroup, multivariate analysis showed that the combined p14^ARF^/p15^INK4b^/p16^INK4a^ status were of independent prognostic significance (Table 3). However, p15^INK4b^, p57^KIP2^ and combined p21^CIP1^/p27^KIP2^/p57^KIP2^ status were not significantly correlated to prognosis (data not shown). We also performed analyses of the subgroup of patients presenting with rest tumor. The parameters used are the same as those used in the analyses of patients who obtained surgical radically (no rest tumor). We were not able to reveal any prognostic significance (data not shown).

## Discussion

Compared to the high level of p57^KIP2^ in normal vulvar squamous epithelium, low p57^KIP2^ expression was identified in 44% of vulvar carcinomas. Low p57^KIP2^ expression has been reported in 89% of non small cell lung cancer [16], 74% of bladder cancer [13], 72% of breast cancer [27], 71% of colorectal carcinoma [23], 60% of ovarian carcinoma [17], 50% of esophageal squamous cell carcinoma [15] and 45% of hepatocellular carcinoma [22], [28]. The wide range of the occurrence of low p57^KIP2^ expression may be due to the various tumor types studied. In our current study, reduced p57^KIP2^ level significantly correlated with a large tumor diameter and increased invasiveness. The decrease in p57^KIP2^ expression with increased tumor size is in line with the findings in some other malignancies, including laryngeal [14], liver [22], oral [29] and pancreatic cancer [30]. Thus the reduced expression of p57^KIP2^ may be involved in progression of different tumors, including vulvar carcinoma.

Previously, homozygous deletion of *p15^INK4b^* has been detected in 3 of 6 (50%) vulvar carcinomas [9]. In the current study, 82% of the vulvar carcinomas had low levels of p15^INK4b^ expression compared to the normal counterpart. The low level of p15 protein in a higher number of vulvar carcinomas than cases with deleted *p15^INK4b^* may reflect that loss of p15 expression are not only due to deletion, but also to mutations and methylation of *p15^INK4b^* gene. Decreased expression of p15^INK4b^ protein has been observed in 54% of malignant peripheral nerve sheat tumor [18]. The *p15^INK4b^* gene has been reported to be homozygously deleted in 50% of T-cell lymphoma [31], 33% of ovarian cancer [19] and 23% of nonsmall cell lung cancer [10], whereas mutations of the *p15^INK4b^* gene were detected in 12% of non small cell lung cancer [10]. In addition, *p15^INK4b^* promoter methylation has been found in 65% of head and neck squamous cell carcinoma [11], 47% of hepatocellular carcinoma [12], 36% of T-cell lymphoma [31] and 30% of ovarian cancer [20]. However, no *p15^INK4b^* deletion and/or mutations and/or methylation were detected in cervical cancer [32] and uveal melanoma [33]. Thus, abnormality of p15^INK4b^ may be tumor specific. Our present results showed a significantly correlation between low p15^INK4b^ expression and malignancy of vulvar carcinomas, including a large tumor diameter and increased invasiveness. Endo et al. [18] have reported in an earlier study of malignant peripheral nerve sheath tumors that protein levels of p15^INK4b^ are significantly lower in large tumors compared with small tumors. Taken together, the low expression of p15^INK4b^ in the majority of vulvar carcinomas and the association with malignant features suggest that p15^INK4b^ may be important in the pathogenesis and/or progression of vulvar carcinomas.

We found no prognostic significance for p57^KIP2^. Similar findings were previously reported in esophageal squamous cell carcinoma [15], colorectal cancer [23], pancreatic carcinoma [30] and ovarian cancer [34]. In contrast, reduced expression of p57^KIP2^ has been correlated with poor outcome in univariate as well as in multivariate analysis in patients with carcinomas of the laryngeal [14] and breast [35]. For patients with ovarian cancer [17] and hepatocellular carcinoma [22] low p57 expression was significantly correlated with poor prognosis in univariate but not in multivariate analysis.

Abnormal expression of p15^INK4b^ has been linked to unfavorable outcome in univariate [19] and multivariate [20] analysis in patients with ovarian cancers, whereas Endo et al. [18] found that in patients with malignant peripheral nerve sheath tumors a decreased expression of p15^INK4b^ was associated with an unfavorable prognosis. We can not confirm the prognostic significance in the present series of vulvar carcinoma patients. However, in both univariate- and multivariate tests a combined analysis of p14^ARF^/p15^INK4b^/p16^INK4a^ showed that patients with tumors expressing low levels of two or three of these INK4 proteins had a worse prognosis than those with only low levels of one or no protein. The same phenomenom has been found in malignant peripheral nerve sheath tumors [18], indicating that a synergetic effect of the combined deficiency for p14^ARF^, p15^INK4b^ and p16^INK4a^ induced high-grade malignancy, not only in malignant peripheral nerve sheath tumors, but also in vulvar carcinomas. The combined analysis of p14^ARF^/p15^INK4b^/p16^INK4a^ may in the future be used as prognostic marker for patients with vulvar carcinomas, but future studies must be performed to eventually confirm a role for these INK4 proteins as predictor for therapy.

In the current study, low p57^KIP2^ expression was significantly associated with low expression of cyclin D3. This is in line with the positive association between p57^KIP2^ and cyclin D1 in esophageal squamous cell carcinoma [15]. In contrast, an inverse correlation has been seen between p57^KIP2^ and cyclin D1 expression in hepatocellular carcinoma [22], whereas such association has not been identified in ovarian cancer [34]. Furthermore, a positive correlation between p57^KIP2^ and cyclin A in colorectal cancer [23] and an inverse association between p57^KIP2^ and cyclin E in ovarian cancer [17] have been reported, which was not found in our study (data not shown). Taken together, these findings suggest that cell cycle proteins regulate the cell growth through different mechanisms in the various tumor types.

## Conclusions

Compared to the high levels of p15^INK4b^ and p57^KIP2^ in normal vulvar squamous epithelium, low levels of p15^INK4b^ and p57^KIP2^ were found in 82% and 44% of vulvar carcinomas, respectively. Furthermore, low levels of p15^INK4b^ and p57^KIP2^ correlated significantly with large tumor diameter and increased invasiveness. These findings suggest that p15^INK4b^ and p57^KIP2^ may be involved in the progression of vulvar carcinomas. Although p15^INK4b^ and p57^KIP2^ levels could not be identified as prognostic markers, combined analysis of p14^ARF^/p15^INK4b^/p16^INK4a^ showed that patients whose tumors expressed low levels of two or three of these INK4 proteins had a worse prognosis than those with only low levels of one or no protein.
